# The prevalence of self-reported deliberate self harm in Irish adolescents

**DOI:** 10.1186/1471-2458-8-79

**Published:** 2008-02-28

**Authors:** Carolyn Morey, Paul Corcoran, Ella Arensman, Ivan J Perry

**Affiliations:** 1 National Suicide Research Foundation, 1 Perrott Avenue, College Road, Cork, Ireland; 2 Inspire Foundation, PO Box 1790, Rozelle NSW 2039, Australia; 3 Department of Epidemiology and Public Health, University College Cork, Room 2.51, Brookfield Health Sciences Complex, College Road, Cork, Ireland

## Abstract

**Background:**

Deliberate self harm is major public health problem, in particular among young people. Although several studies have addressed the prevalence of deliberate self harm among young people in the community, little is known about the extent to which deliberate self harm comes to the attention of medical services, the self harm methods used and the underlying motives. The aim of this study was to determine the prevalence of deliberate self harm in adolescents and the methods, motives and help seeking behaviour associated with this behaviour.

**Methods:**

A cross-sectional survey using an anonymous self-report questionnaire was administered in 39 schools in the Southern area of the Health Service Executive, Ireland. Of the 4,583 adolescents aged 15–17 years who were invited to participate in the survey, 3,881 adolescents took part (response: 85%).

**Results:**

A lifetime history of DSH was reported by 9.1% (n = 333) of the adolescents. DSH was more common among females (13.9%) than males (4.3%). Self cutting (66.0%) and overdose (35.2%) were the most common DSH methods. A minority of participants accessed medical services after engaging in DSH (15.3%).

**Conclusion:**

DSH is a significant problem in Irish adolescents and the vast majority do not come to the attention of health services. Innovative solutions for prevention and intervention are required to tackle DSH in adolescents.

## Background

Deliberate self harm (DSH) is a major public health problem in Ireland. Young people are those most at risk, particularly young women aged 15–19 years who had the highest incidence of DSH based on hospital presentations to A&E departments in 2004, 606 per 100,000[1]. While the rate for young men of the same age is considerably lower, it is also notable at 301 per 100,000[1]. These incidence rates are likely to be the 'tip of the iceberg' as they do not include cases who do not present to hospital after an act of DSH. *Deliberate self harm *is used to describe different types of behaviours associated with varying levels of suicide intent and a variety of motives.

The self reported lifetime prevalence of DSH found by general population studies of adolescents ranges from 2–8%[2-4]. Two Irish school-based studies have reported a lifetime prevalence of DSH in adolescents. In a sample of 88 13–14 year-olds a lifetime prevalence of self harm of 2–3% was reported [5], while Lynch and colleagues found a lifetime prevalence of deliberate self harm of 1.5% among adolescents aged 12–15 years [6]. In two studies, the self reported life time prevalence of self harm thougths was reported, which was 44% [5] and 45.7%[7].

This paper reports the findings of a large-scale school-based survey in Ireland that investigated the prevalence of DSH and self harm thoughts in 15–17 year olds. Details of the methods of DSH, motives for DSH and help seeking behaviour are addressed as well.

## Methods

### Design and sample

A cross-sectional survey of 3,881 adolescents aged 15–17 years was carried out in 39 schools in the Health Service Executive (HSE) Southern area between January 2003 and March 2004. Power calculations indicated that a minimum of 3,000 students were required to return a 95% confidence interval of 9.0–11.0% for a postulated prevalence of 10% as reported by Hawton and colleagues[8]. A list of all schools within the Southern area of the Health Service Executive was obtained from the Department of Education and schools were then divided according to whether they were in Counties Cork or Kerry or Cork city. They were further categorised as being co-educational, all male or all female. Using a random selection, 54 schools were invited to take part and 39 schools participated in the survey. Of the 4,583 students who were invited to take part, 3,881 participated in the survey, which is a response rate of 85%. Fifty one questionnaires (1.3%) were excluded from data analysis as they did not fit the age criteria (n = 25) or were not filled in seriously (n = 26).

The response rate varied by school. For 7 of the 39 schools, the response rate was lower than 75% whereas 8 schools had a response rate of at least 95%. However, there was no strong association between type of school and response rate. Non-participation was primarily because of absenteeism. In total, 90.7% of the non-responding children were absent. Those absent due to illness or deliberately not coming to school would have a health and social profile that would be associated with a higher prevalence of DSH to those in school. However, students were also absent from school because of out-of-school activities such as day trips and tours etc. This was the primary reason why some schools had a relatively poor response rate. Such students would be likely to have a similar prevalence of DSH to those in school at the time of the survey.

With regard to the participating and non-participating schools, single-sex girls' schools were slightly overrepresented among participating schools but no differences were found in terms of city/county location.

### Procedure

Prior to the survey being completed, principals and teaching staff were informed about the procedure. An information sheet and opt out form were also sent to parents. On the day when the survey was carried out, students were also given the opportunity to opt out. The Clinical Research Ethics Committee of the Cork Teaching Hospitals granted ethical approval for this survey.

This survey was part of a European survey known as the Child and Adolescent Self Harm in Europe (CASE) study, which has been administered in 6 countries across Europe and one centre in Australia. A standardised internationally validated, anonymous questionnaire was designed by CASE collaborators and used for data collection by each of the centres.

The questionnaire was administered with a member of the research team being present and completed by students at school in the class room. An introduction explained the anonymity and confidential nature of the data along with the voluntary nature of students' participation. It was clarified to the students that they were free to choose whether to complete any or all of the questionnaire and their choice had no bearing on their school work. Completion of the questionnaire took 20–30 minutes. After participants had completed the survey there was a general discussion about the help and support available for young people in their local communities and each participant received a resource pack, which included a list of services in their local area. Time was made available immediately after the session for individuals who wished to ask further questions. There was also the option of contacting the National Suicide Research Foundation at any time if further assistance was required.

A distinctive characteristic of this study was that participants were asked to describe in their own words, the method(s) they had used to harm themselves. This description was then coded according to a standardised definition of deliberate self harm. The definition of deliberate self harm is as follows: "An act with a non-fatal outcome in which an individual deliberately did one or more of the following:

• Initiated behaviour (for example, self cutting, jumping from a height), which they intended to cause self harm.

• Ingested a substance in excess of the prescribed or generally recognisable therapeutic dose.

• Ingested a recreational or illicit drug that was an act that the person regarded as self harm.

• Ingested a non-ingestible substance or object."

This definition includes acts that are interrupted before self harm is inflicted, for example, a person removed from a bridge before jumping off or interrupted attempts of hanging, but excludes episodes of self harm by individuals who do not understand the meaning or the outcome of their act, for example due to a learning disability. The following questions were used to identify deliberate self harm: *"Have you ever deliberately taken an overdose (e.g. pills or other medication) or tried to harm yourself in some other way (such as cut yourself)?" *with response options:*No/Yes, once/Yes, more than once*. For those who confirmed having engaged in DSH, the questionnaire included further questions about the timing of the last act of DSH *(less than a month ago/between a month and a year ago/more than a year ago)*, and participants were asked to describe details of the self harm act, for example the name of the drug taken in an overdose.

Episodes of deliberate self harm were then classified as a 'yes', 'no' or 'no information given' by three independent raters (CM, PC, EA) using the standardised criteria (Cohen's Kappa = 0.77). In cases where ratings were inconsistent, decisions were made based on majority rating. For those who indicated they had harmed themselves, the questionnaire included a series of questions relating to motives, methods and help seeking behaviour.

Self harm thoughts were examined by the following question: *"Have you during the past month or the past year seriously thought about taking an overdose or trying to harm yourself but not actually done so?" *with response options: *No/Yes, the last time was in the past month/Yes, the last time was over a month ago, but less than a year ago*. For those who reported self harm thoughts, the questionnaire included further questions related to help seeking behaviour.

### Data analysis

Prevalence rates of deliberate self harm and self-harming thoughts were calculated with adjustment for missing data. For the prevalence rates, 95% binomial confidence intervals were calculated. Relative risks and their associated 95% confidence intervals were used to assess the differences in prevalence rates between groups.

## Results

### Demographics

Of the 3,830 questionnaires, 50.2% were females and the majority (53.1%) of students were 16 years of age (Table 1). The gender balance of the sample and the proportion attending schools in Cork City and Counties Kerry and Cork were similar to the distribution of the adolescent population in the region. There was a difference in the age distribution as the sample was centred on 16 year-olds. This age accounted for just over half of the sample while the remainder were evenly made up of 15 and 17 year-olds. The general population of 15–17 year-olds were made up of almost equal proportions at each year of age.

**Table 1:** Description of sample and prevalence of DSH by sociodemographic factors

		**Number (%)**	**Prevalence of DSH (95% CI)**
**Gender**	Male	1902 (49.8%)	4.4% (3.1 – 5.6%)
	Female	1915 (50.2%)	13.9% (11.8 – 16.1%)

**Age**	15 years	884 (23.2%)	9.3% (7.5 – 11.2%)
	16 years	2021 (53.1%)	9.4% (7.6 – 11.2%)
	17 years	902 (23.7%)	8.5% (6.8 – 10.3%)

**City/County**	Cork City	996 (26.0%)	9.4% (7.6 – 11.2%)
	Cork County	1965 (51.3%)	9.1% (7.3 – 10.9%)
	Kerry County	869 (22.7%)	8.9% (7.1 – 10.6%)

**Living arrangement**	Both parents	3224 (84.6%)	8.1% (6.4 – 9.8%)
	One parent	442 (11.6%)	14.3% (12.1 – 16.4%)
	One parent and a step parent/partner	100 (2.6%)	18.1% (15.7 – 20.5%)
	Other family member	23 (0.6%)	15.0% (12.8 – 17.2%)
	Other	22 (0.6%)	10.5% (8.6 – 12.4%)

### Deliberate self harm

A lifetime history of self reported deliberate self harm was reported by 458 (12.2%, 95% CI: 11.2%-13.3%) of the teenagers surveyed (Table 2). Of the total population, 333 (9.1%, 95% CI: 8.2% – 10.1%) students met the DSH definition. Of those who had harmed themselves, 45.9% had done so more than once. There were 807 (21.6%, 95% CI: 20.3%-23.0%) teenagers who reported thinking about harming themselves in the previous year, with girls reporting self harm thoughts twice as likely than boys (29.9% vs. 13.2%, Relative Risk (RR) = 2.3, 95% CI: 1.9- 2.6).

**Table 2:** Prevalence of deliberate self harm

	**Self report^1^**		**Based on standardised DSH description^2^**	
	**N/total respondents**	**% (95%CI)**	**N/total respondents**	**% (95%CI)**
Lifetime	458/3747	12.2% (11.2%-13.3%)	333/3646	9.1% (8.2% – 10.1%)
- More than a year ago	173/3747	4.6% (4.0%-5.4%)	117/3646	3.2% (2.7% – 3.9%)
- Past year	266/3747	7.1% (6.3% – 8.0%)	208/3646	5.7% (5.0% – 6.5%)
- Past month	83/3747	2.2% (1.8% – 2.7%)	65/3646	1.8% (1.4% – 2.3%)
**Students who had thought of harming themselves but did not go through with it**				
	**n/total respondents**	**% (95%CI)**		

Past Year	807/3732	21.6% (20.3% – 23.0%)		
Past Month	313/3732	8.4% (7.5% – 9.3%)		

^1 ^19 did not specify the time of their last DSH act

^2 ^8 did not specify the time of their last DSH act

### DSH and sociodemographic factors

Girls were three times more likely to harm themselves than boys (RR = 3.2 95 % CI: 2.5 – 4.1; Table 1). There was no statistically significant variation with regard to age and similar prevalences of DSH were reported by the students of schools in Cork city and the counties of Cork and Kerry. Living arrangement was associated with significant variation in the prevalence of DSH. Living with both parents was protective. Relative to teenagers with this living arrangement, DSH was significantly more common among those living with one parent (RR = 1.8 95 % CI: 1.4 – 2.3) or one parent and a step parent/partner (RR = 2.2 95 % CI: 1.4 – 3.4).

### Methods of deliberate self harm

The majority of the teenagers who engaged in self harm did so by either cutting themselves (66.0%) or taking an overdose (35.2%) (Table 3). Although there was a higher proportion of girls who reported cutting themselves or taking an overdose than boys, this was not significant. It is notable that boys used a greater variety of methods to harm themselves than girls. Of the 333 teenagers who had harmed themselves, 19.8% did so under the influence of alcohol, while 11.8% were under the influence of an illegal drug at the time when they harmed themselves. Boys (32.3%) were twice as likely as girls (15.4%) to have been under the influence of alcohol when they engaged in DSH (RR = 2.1, 95% CI: 1.3 – 3.4) and four and half times more likely to have taken an illegal drug (28.1% V 6.1%, RR = 4.6, 95% CI: 2.2 – 9.5). Of the 220 cases of self-cutting, 11.5% also involved an overdose.

**Table 3:** Methods of deliberate self harm by gender

	**Self cutting**	**Overdose**	**Alcohol**	**Recreational Drugs**	**Self Battery**	**Hanging**	**Other**	**Total**
**Girls**	174 (68.8%)	89 (35.2%)	10 (4.0%)	5 (2.0%)	7 (2.8%)	5 (2.0%)	11 (4.3%)	253
**Boys**	45 (57.0%)	19 (24.1%)	6 (7.6)	8 (10.1%)	5 (6.3%)	4 (5.1%)	4 (5.1%)	79
**Total**	220* (66.0%)	108 (32.4%)	16 (4.8%)	13 (3.9%)	12 (3.6%)	9 (2.7%)	15 (4.5%)	333*

*Gender was unknown in 1 case of self-cutting

**Please note**: Percentages add up to more than 100 as multiple methods of DSH are reported

### Motives for deliberate self harm

The two most common motives reported by students for DSH were; *"I wanted relief from a terrible situation" *(78.9%) and *"I wanted to die" *(60.9%) (Table 4). Boys more often reported wanting to frighten someone as a motive for their DSH than girls (40.7% vs. 23.6%, RR = 1.7, 95% CI: 1.1 – 2.6). Also, boys were somewhat more likely to report that they wanted to find out if someone really loved them as a motive for there DSH (38.2% vs. 25.8%, RR = 1.5 95% CI: 1.0 – 2.2). Of the 333 students who harmed themselves, nobody reported wanting to die as the only motive for DSH.

**Table 4:** Motives reported for deliberate self harm by gender

	**Females **(N = 253) **%**	**Males **(N = 79) **%**	**Relative Risk (M/F)**	**95% CI**
*Wanted to get relief from a terrible state of mind*	79.2	77.6	1.0	0.8 – 1.1
*Wanted to die*	59.7	65.0	1.1	0.9 – 1.3
*Wanted to show how desperate I was feeling*	52.1	52.8	1.0	0.7 – 1.3
*Wanted to punish myself*	43.8	49.1	1.1	0.8 – 1.5
*Wanted to find out whether someone really loved me*	25.8	38.2	1.5	1.0 – 2.2
*Wanted to frighten someone*	23.6	40.7	1.7	1.1 – 2.6
*Wanted to get some attention*	23.0	27.3	1.2	0.7 – 1.9
*Wanted to get my own back on someone*	19.7	25.0	1.3	0.7 – 2.2

### Help seeking behaviour among those who harmed themselves

Forty four percent (43.7%, 95% CI: 40.8% – 47.0%) of teenagers who harmed themselves sought help prior to them engaging in the DSH, while 49.8% (95% CI: 46.9% – 53.1%) sought help after the event. Of those seeking help, contacting a friend was the most common source of help sought both before (38.2%) and after (40.3%) engaging in DSH (Table 5). Teenagers were more likely to contact health services after they had harmed themselves rather than before, likewise family members were more likely to be aware of the self harm after the event. Of those who harmed themselves, only 36 (11.3%) reported that they presented to hospital as a result of their DSH episode.

**Table 5:** Help seeking behaviour among those who harmed themselves (n = 333)

Source of help	Before DSH	After DSH
Family	7.8%	20.5%
Friend	38.2%	40.3%
Teacher	5.8%	6.5%
GP	1.8%	7.7%
Social Worker	3.3%	4.5%
Psychologist/Psychiatrist	4.0%	9.2%
Telephone Helpline	4.4%	1.6%
Drop-in/Advice Centre	1.1%	1.2%
Other	11.2%	7.1%

## Discussion

In this broad representative sample of adolescents aged 15–17 years approximately one in ten reported a previous episode of DSH of whom 45.9% had done so more than once. The lifetime prevalence of DSH of 9.1% is higher compared to the prevalence of deliberate self harm reported in previous Irish school-based studies [5-7], which can be explained by the use of a broader definition of self harming behaviour in the present study. Only 11.3% of teenagers who harmed themselves attended hospital and fewer sought help from other medical services following their episode of DSH, findings which are consistent with other Irish studies [9,10]. Given this tendency for adolescents not to seek help and that most studies investigating deliberate self harm are based on hospital treated cases of deliberate self harm [11], previous estimates of the extent of deliberate self harm may have been underestimated.

The one-year prevalence rate based on the study definition criteria was 5.7%. Considering their lifetime history of DSH, 11.3% of the teenagers had attended hospital at least once. This figure reduced to 8.3% when only considering cases of DSH that occurred in the past year. Combining the one-year prevalence and its associated figure for hospital attendance indicated that the annual incidence of hospital-treated adolescent DSH in the region was 473 per 100,000. This is 54% higher than the rate of 307 per 100,000 calculated for 15–17 year-olds of the region based on data from the National Registry of Deliberate Self Harm [1]. Possible reasons for this discrepancy include the broader definition used in the school-based study, adolescents underestimating the time to their previous DSH, and DSH episodes going undetected within the hospital system.

Unlike many other self-report studies examining deliberate self harm, in this study a standardised definition of deliberate self harm was used, thereby enabling valid international comparisons with other European countries. The lifetime prevalence of DSH among adolescents in the different regions participating in the CASE study ranged from 4.1% in The Netherlands [12] to 13.2% in the United Kingdom [8]. In this context, the lifetime DSH prevalence of 9.1% among Irish school going adolescents can be considered relatively high. In comparison with prevalence rates reported in international studies using slightly different methodologies and focusing on a broader age range among adolescents, again the prevalence of DSH in Irish adolescents appears to be higher. In a cross-sectional survey among 12 to 15 year olds in the U.S state of Washington, Patton et al. found that 3.7% reported an episode of DSH in the 12 months prior to the survey, which is lower than the 12 month DSH prevalence (5.7%) in the present study [13]. In a general population based survey in Norway among 13–19 year olds, Groholt et al found a life time DSH prevalence of 8.1%, which is fairly similar to the Irish prevalence [4].

One drawback to the use of this definition is that it is likely to have produced a conservative estimate of deliberate self harm amongst Irish adolescents due to the requirement of validating response to the initial DSH question by a detailed description. In the present study, 104 participants responded positive to the initial DSH question, but did not provide a detailed description of the self harm act, and were therefore excluded from the analyses.

The current study shows that teenage girls are significantly more likely to harm themselves than teenage boys, which is consistent with a similar study in England. Adolescent girls aged 15 and 16 years were 3.5 times more likely (11.2% vs. 3.2%) to engage in self harm than males of the same age[8]. While this finding has important implications for designing prevention and intervention programs for preventing deliberate self harm and promoting positive mental health, it is important not to neglect adolescent boys as they appear to have the highest risk of taking their life and they are also less likely to talk to someone or seek help if they do have problems[14,15].

Self-cutting was the most common method of deliberate self harm reported by both males and females. This is different compared to the hospital presentations due to DSH by adolescents as monitored by the National Registry of Deliberate Self Harm, where overdose appeared to be the most common method of deliberate self harm[1].

Our findings in relation to the motives for DSH are in line with findings from other studies[16-18]. The most frequently reported motives in our study included, *'wanting to get relief from a terrible situation' *(78.9%) and *'wanting to die' *(60.9%). It is notable that although *'wanting to die' *was the second most frequently reported motive, no participant reported this motive as a single motive for DSH, which reflects an ambivalent attitude whereby the expression of feelings of extreme anxiety that the adolescent wishes to escape from prevail. These findings also support the notion that not all instances of DSH are motivated by suicidality.

## Conclusion

In conclusion, there is an urgent need to address the issue of deliberate self harm amongst Irish adolescents. Programmes and interventions that raise awareness about positive mental health and help seeking behaviours should be considered. Along with health services finding ways to eliminate the barriers felt by adolescents that may prevent them from seeking help and to make contact with adolescents in a youth friendly medium rather than waiting for adolescents to come to health services.

## Competing interests

The author(s) declare that they have no competing interests.

## Authors' contributions

CM drafted the manuscript and was involved in the data collection and data analysis. PC and IJP were involved in the design of the study. CM and EA were involved in the coordination of the data collection. PC had a leading role in the data analysis. All authors were involved in the interpretation and writing of the final manuscript.

## Pre-publication history

The pre-publication history for this paper can be accessed here:


